# Explanatory model of behavioral adaptation in video game addiction among adolescents in urban and rural Peru: the mediating role of anxiety

**DOI:** 10.3389/fpsyg.2025.1500800

**Published:** 2025-04-08

**Authors:** Julio Cesar Huamani-Cahua, Estefany Ojeda-Flores, Norma Roxana Medina Arce, Leslie Emilia Villanueva Kuong, Michael Antony Ojeda-Flores

**Affiliations:** ^1^Graduate School of the Universidad Católica de Santa María, Arequipa, Peru; ^2^Universidad Continental, Huancayo, Junin, Peru; ^3^Department of Psychology, Universidad Tecnológica del Peru, Lima, Peru

**Keywords:** behavioral regulation, video game dependency, state anxiety, trait anxiety, mediation

## Abstract

**Introduction:**

The study aimed to determine the relationships between behavioral adaptation and video game addiction, mediated by anxiety, in Peruvian adolescents from urban and rural areas, using a structural equation modeling (SEM) approach.

**Methods:**

This explanatory and cross-sectional study employed convenience sampling, comprising 606 students of both sexes, aged 11 to 13, with 62.4% from urban areas and 37.6% from rural areas. The instruments used included the State-Trait Anxiety Inventory for Children (STAIC) to measure state and trait anxiety, the Behavioral Adaptation Inventory (IAC), and the Video Game Dependency Test (TDV). These instruments demonstrated adequate validity and reliability for the sample through confirmatory factor analysis (CFA), ensuring their relevance in the Peruvian context.

**Results:**

The SEM results confirmed that behavioral adaptation influences video game addiction, mediated by anxiety, with good model fit indices (χ^2^/df = 4.836; TLI = 0.945; CFI = 0.964; GFI = 0.950; RMSEA = 0.080, 90% CI [0.068, 0.092]). Regarding anxiety types, state anxiety showed a stronger negative mediating effect (*β* = −0.31; *β* = 0.20) compared to trait anxiety (*β* = −0.22; *β* = 0.16). Significant differences were found between rural and urban students, with rural adolescents exhibiting lower behavioral adaptation and higher levels of state and trait anxiety (*p* < 0.001) compared to their urban peers.

**Discussion:**

The findings support theories emphasizing the interaction between emotional and behavioral factors in the development of problematic behaviors. Additionally, state anxiety is identified as having a greater mediating impact than trait anxiety, suggesting that situational emotional responses, rather than stable predispositions, are key determinants in intensifying addictive behaviors in specific contexts.

## Introduction

Video games are one of the most popular entertainment media worldwide, particularly among adolescents, who use them as a form of leisure and socialization that impacts their values and behaviors (Limone et al., 2023; Rodríguez and Padilla, 2021; Guzmán-Brand and Gelvez-Garcia, 2022).

While playing video games can have positive effects on adolescents (Raith et al., 2021; Liao et al., 2023; Portillo Peñuelas et al., 2023), problematic use or addiction may lead to violent behavior, family conflicts, and emotional, academic, and social difficulties (Luo et al., 2022; Chen et al., 2025; Wang et al., 2020; Estrada-Araoz et al., 2023; González Moreno and Molero Jurado, 2022; Hou et al., 2022; Jadallah et al., 2023; Limone et al., 2023; Mbarki et al., 2023).

Video game addiction involves compulsive dependence on gaming, characterized by a lack of control over its use, prioritizing gaming over relationships, responsibilities, or personal goals (Estrada-Araoz et al., 2023; Raney et al., 2023; Mohamed et al., 2023).

Globally, an increasing trend of adolescents showing risk criteria associated with problematic video game use has been observed (Long et al., 2018; Colasante et al., 2022; Mohamed et al., 2023; Raney et al., 2023; Chen et al., 2025). In Peru, studies such as Király et al. (2019) reported high levels of this problem, findings corroborated by Estrada-Araoz et al. (2023).

The classification of video game disorder is relatively recent. The inclusion of “video game addiction” in the DSM-5 marked its formal recognition as a behavioral addiction (American Psychiatric Association, 2013; Luo et al., 2022). The ICD-11 also recognized this disorder, providing specific diagnostic criteria (Costa and Kuss, 2019; Dong et al., 2019; Luo et al., 2022). Although there appears to be consensus on the harmful effects of this addiction, the need for further research persists (American Psychiatric Association, 2013; Costa and Kuss, 2019) to deepen the understanding of this phenomenon, particularly the processes contributing to its development (Dong et al., 2019; Von der Heiden et al., 2019; Brand et al., 2020).

For adolescents, behavioral adaptation is crucial for development, enabling them to adjust to environmental demands. This process includes identifying resources, interpreting norms, and adapting expectations and behaviors to social dynamics (Bandzeladze et al., 2019; Silvers, 2022; Li et al., 2023; Choe et al., 2024).

Inadequate adaptation can hinder the management of interpersonal relationships, academic demands, and family conflicts, which could increase an individual’s vulnerability to problematic behaviors (Artuch-Garde et al., 2017; Ruiz and Yabut, 2024), such as video game addiction (Wang and Cheng, 2022; Lin et al., 2024; Chen et al., 2025).

### Anxiety as a mediator

Adaptation difficulties are often associated with increased anxiety levels, a common adaptive response that allows individuals to face challenges (del Río Olvera et al., 2018; Dolz-del-Castellar and Oliver, 2021; Kurth and Pihkala, 2022; Mishu et al., 2023).

Although it has been hypothesized that anxiety could play the role of a mediator between behavioral adaptation and the development of video game addiction in adolescents, it had not yet been clearly established which type of anxiety is associated with such addiction. Brand et al. (2014) highlight the importance of personal factors, including biological ones, such as trait anxiety, which refers to a consistent predisposition to experience anxiety in various contexts, even when it is not justified (Fujihara et al., 2023; Portela-Pino and Dominguez Rodriguez, 2023). This trait could be a key factor in the accelerated development of addiction. However, the same authors, in clarifying and improving their model (Brand, 2017), emphasize the relevance of cognitive and emotional factors as fundamental elements in this process.

In this regard, it would be more appropriate to focus on state anxiety, referring to the temporary condition in which a person experiences high levels of anxiety (Dolz-del-Castellar and Oliver, 2021; Fujihara et al., 2023; Portela-Pino and Dominguez Rodriguez, 2023). In line with this, some studies, such as that of Mehroof and Griffiths (2010), support the hypothesis that state anxiety is more significant. Therefore, one of the objectives of this study will be to evaluate which of these forms of anxiety has a more substantial influence on the sample.

These anxiety level changes could relate to a greater tendency toward disorders or addictions as forms of negative coping and avoidance (Colder Carras et al., 2017; Huang et al., 2022). While no studies integrate all these variables, prior research has identified a significant relationship between anxiety and addiction development (Von der Heiden et al., 2019; Huang et al., 2022; Qin et al., 2023). Similarly, individual factors such as adaptation difficulties could be intensified by affective elements like anxiety, fostering the emergence of problematic behaviors (Adams et al., 2019; Király et al., 2023; Li et al., 2023).

The I-PACE theoretical model (Interaction of Person-Affect-Cognition-Execution), developed by Brand et al. (2014), provides a comprehensive framework for understanding behavioral addictions, including video game addiction (Brand, 2017; Dong et al., 2019; Brand et al., 2020). This model highlights that addictions arise from dynamic interactions between personal, affective, cognitive, executive, and situational factors (Brand et al., 2014; Brand, 2017; Dong et al., 2019; Mehmood et al., 2021). In this study, the I-PACE model is relevant because behavioral adaptation, as a personal factor, and anxiety, as an affective factor, could influence the processes leading to video game addiction.

In Peru, most research on video game addiction focuses on its effects or correlations with isolated variables (Salas-Blas et al., 2016; Mamani and Yupanqui, 2018; Király et al., 2019; Cabrejos and Flores, 2020; Aguirre Linares and Verdi Matto, 2022; Hernández-Vásquez et al., 2022; Ramos Fernández and Pérez Silva, 2022; Estrada-Araoz et al., 2023; Moya et al., 2023; Sairitupa-Sanchez et al., 2023; Tacanga Polo and Ulloa Vargas, 2024). This limited focus highlights the need for studies identifying predictive variables for this behavior, especially considering alarming mental health figures among Peruvian adolescents.

This study proposes analyzing the relationships between behavioral adaptation and video game addiction mediated by anxiety in Peruvian adolescents using a SEM approach. This methodology allows exploration of direct and indirect relationships between these variables, offering an integrative perspective relevant to the national context, where research remains limited.

Based on this theoretical framework, the following hypotheses were proposed:

*H1*: Behavioral adaptation influences anxiety in Peruvian adolescents in urban and rural areas.

*H2*: Behavioral adaptation influences video game addiction in Peruvian adolescents in urban and rural areas.

*H3*: Anxiety influences video game addiction in adolescents from urban and rural areas.

*H4*: Behavioral adaptation mediated by anxiety has an indirect positive effect on video game addiction.

*H5*: State anxiety has a greater mediating effect than trait anxiety in the relationship between behavioral adaptation and video game addiction.

### Variables studied

Anxiety: state anxiety, trait anxiety

Behavior adaptation

Video game addiction

**Figure 1:**
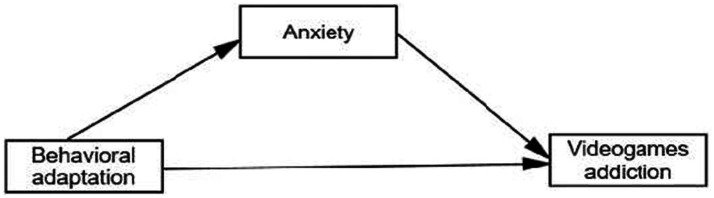
Theoretical explanatory model of video game addiction. Explanatory model of video game addiction.

Area: Urban (1), rural (2) (Figure 1).

## Methodology

### Design

An explanatory, cross-sectional design was used to investigate the functional relationships between two or more variables by predicting a criterion variable using one or more predictors at a specific point in time (Ato et al., 2013).

a. Regression of X (Independent variable: Behavioral adaptation) and M (mediator: Anxiety)

M = β_o_ + β_1_X_1_ + e.

b. Regression between variable X (Independent variable: Behavioral adaptation) and Y (Dependent variable: video game addiction).

Y =  β_o_ + β_1_X_1_ + e.

c. Regression between X (independent variable: behavioral adaptation) and M (anxiety), on Y (dependent variable: Video game addiction).

Y = β_o_ + β_1_X_1_ + β_2_X_2_ + e.

Assumptions (Baron and Kenny, 1986):

1. X must have a significant effect on M.

2. X must have a significant effect on Y.

3. M must have a significant effect on Y.

### Participants

A convenience sampling method was employed, based on accessibility and proximity to the researcher. The sample consisted of 606 students aged 11–13 years (*M* = 12.11; *SD* = 0.69), 51.8% female and 48.2% male, with 62.4% from urban areas (four schools) and 37.6% from rural areas (five schools).

### Instruments

The Behavioral Adaptation Inventory (IAC) by Victoria de la Cruz and Agustín Cordero, published by TEA Ediciones in Spain (1981, 2004, first and sixth editions), adapted in Peru by Ruiz (1995), consists of 123 items with Yes, No, and? responses. It measures four areas: personal, family, educational, and social. In Peru, the IAC has shown adequate criterion validity values when correlated with Bell’s Adaptation Questionnaire for Adolescents (Bell, 1934), adapted by Cerdá (1987). Additionally, it demonstrates good validity (CFA) and reliability indices (Asto, 2016). For this study, internal structure validity was confirmed, with acceptable goodness-of-fit indices (χ^2^/df = 1.59; CFI = 0.944; TLI = 0.943; RMSEA = 0.031, 90% CI [0.030, 0.032]; SRMR = 0.104). Reliability was evaluated using Cronbach’s alpha, with good and acceptable values for Personal Adaptation (*α* = 0.892; 95% CI [0.871, 0.898]), Family Adaptation (α = 0.669; 95% CI [0.621, 0.704]), Educational Adaptation (*α* = 0.788; 95% CI [0.763, 0.811]), and Social Adaptation (*α* = 0.714; 95% CI [0.680, 0.746]).

The State–Trait Anxiety Inventory for Children (STAIC) by Spielberger (1973), adapted to Spanish by Seisdedos (1990), includes two separate scales: one for assessing State Anxiety (A-E) with 20 items measuring anxiety at a specific moment, and another for Trait Anxiety (A-R) with 20 items identifying general feelings. Designed for children aged 8 to 15 years, it uses a three-point Likert scale (1 = “nothing,” 2 = “somewhat,” and 3 = “a lot”), with higher scores indicating higher anxiety levels. The instrument shows good validity and reliability indices in the Peruvian sample (Céspedes, 2015). In this study, CFA confirmed internal structure validity for State and Trait Anxiety. For State Anxiety, the model by Calane (2022) was confirmed, showing good fit indices (χ^2^/df = 2.46; CFI = 0.914; TLI = 0.903; RMSEA = 0.049, 90% CI [0.043, 0.055]; SRMR = 0.070). For Trait Anxiety, the unidimensional model was validated, with robust fit indices (χ^2^/df = 1.69; CFI = 0.977; TLI = 0.974; RMSEA = 0.032, 90% CI [0.026, 0.039]; SRMR = 0.043). Reliability indices were good for affirmative State Anxiety (*ω* = 0.732), negative State Anxiety (ω = 0.735), and Trait Anxiety (ω = 0.788).

The Video Game Dependency Test (TDV) by Chóliz and Marco (2011), based on DSM-IV-TR criteria, originally consisted of 55 items. Following validation by judges, it was reduced to 32 items and further refined to 25 items after psychometric analysis. Responses are rated on a scale from zero (0) to four (4), ranging from “strongly disagree” to “strongly agree.” The original instrument shows solid internal consistency (*α* = 0.94) and construct validity with four factors: Withdrawal, Abuse/Tolerance, Problems Caused by Video Games, and Difficulty Controlling Use. In Peru, Salas-Blas et al. (2016) validated the instrument for adolescents aged 11–18 years in Lima, with a linguistic adaptation to assess comprehension. CFA for this study confirmed the internal structure validity of the four-factor model, with good fit indices (χ^2^ = 716.846; df = 269; χ^2^/df = 2.665; CFI = 0.974; TLI = 0.971; RMSEA = 0.052, 90% CI [0.048, 0.057]; SRMR = 0.047). Reliability indices were also adequate: Withdrawal (ω = 0.888), Abuse/Tolerance (ω = 0.732), Problems Caused by Video Games (ω = 0.777), and Difficulty Controlling Use (ω = 0.778).

### Procedures

The process began with obtaining permissions from school directors. Detailed arrangements were made to establish convenient schedules for administering the instruments in the school environment. Training sessions were conducted to ensure uniformity in instrument application and comprehension of procedures. The next step involved obtaining informed consent and assent from participants, ensuring their understanding and voluntary agreement. With authorization secured, instruments were administered following established protocols.

### Data analysis

The analysis was conducted in three stages. The first stage involved validating and confirming the internal structure of the Behavioral Adaptation Inventory (IAC), the State–Trait Anxiety Inventory for Children (STAIC), and the Video Game Dependency Test (TDV) by Chóliz and Marco (2011) using Confirmatory Factor Analysis (CFA) with the statistical software R and the *lavaan* package in RStudio (R Core Team, 2023), employing the WLSMV estimator. Goodness-of-fit indices such as CFI, TLI, SRMR, and RMSEA were evaluated. In the second stage, reliability was determined using JASP software (JASP Team, 2024), applying McDonald’s Omega coefficient, which is suitable for factorial models (Hayes and Coutts, 2020), and Cronbach’s Alpha coefficient (Taber, 2018). In the third stage, AMOS version 21 was used to analyze the explanatory model through Structural Equation Modeling (SEM). The exogenous variables were Behavioral Adaptation and Anxiety, while the latent endogenous variable was Video Game Addiction, confirming the theoretical model. The maximum likelihood method was applied, as well as Harman’s test (Podsakoff et al., 2003) for common variance. The evaluation criteria considered were CFI, TLI, and GFI with values ≥0.90 (Bentler, 1990; Hu and Bentler, 1999), RMSEA with values ≤0.08 (MacCallum et al., 1996), χ^2^/df < 5 (Osborne and Hancock, 2008), and standardized coefficients >0.1 (Barclay et al., 1995).

### Ethics statement

The study complies with ethical standards, ensuring voluntariness, confidentiality, and respect for participants’ rights. Participation of minors was conditioned on informed consent from their guardians, who received detailed explanations of the research’s objectives and scope. Collected data were coded numerically to ensure anonymity and used exclusively for research purposes. Guardians retained the right to withdraw consent and their child’s participation without negative repercussions. Data handling adhered to the Habeas Data Law, and the project was approved by a University Ethics Committee (Favorable Opinion No. 157–2024). Full informed consent is included in the ethics committee’s report annex.

## Results

Table 1, indicates relationships between the model variables and video game addiction. Overall, results show that mean levels of behavioral adaptation (*M* = 60.68, *SD* = 17.61), state anxiety (*M* = 32.79, *SD* = 5.22), and trait anxiety (*M* = 33.57, *SD* = 5.86) are relatively low. Similarly, the average score for video game dependency (*M* = 22.67, *SD* = 20.17) suggests mild rather than severe dependency. Correlations confirm these relationships: behavioral adaptation negatively correlates with state anxiety (*r* = −0.311) and trait anxiety (*r* = −0.218), while both types of anxiety positively correlate with video game dependency (*r* = 0.252 for state anxiety, *r* = 0.221 for trait anxiety).

**Table 1:** Descriptive statistics and correlations of variables.

Variables	M	SD	A	1	2	3	4	5
1. Age	12.11	0.69	−0.15	1				
2. Behavioral adaptation	60.68	17.61	−0.17	−0.150^**^	1			
3. State anxiety	32.79	5.22	0.41	0.100^*^	−0.311^**^	1		
4. Trait anxiety	33.57	5.86	0.20	0.018	−0.218^**^	0.328^**^	1	
5. Video game addiction	22.67	20.17	0.70	0.073	−0.189^**^	0.252^**^	0.221^**^	1

*p <0.05; **p< 0.01.

**Figure 2:**
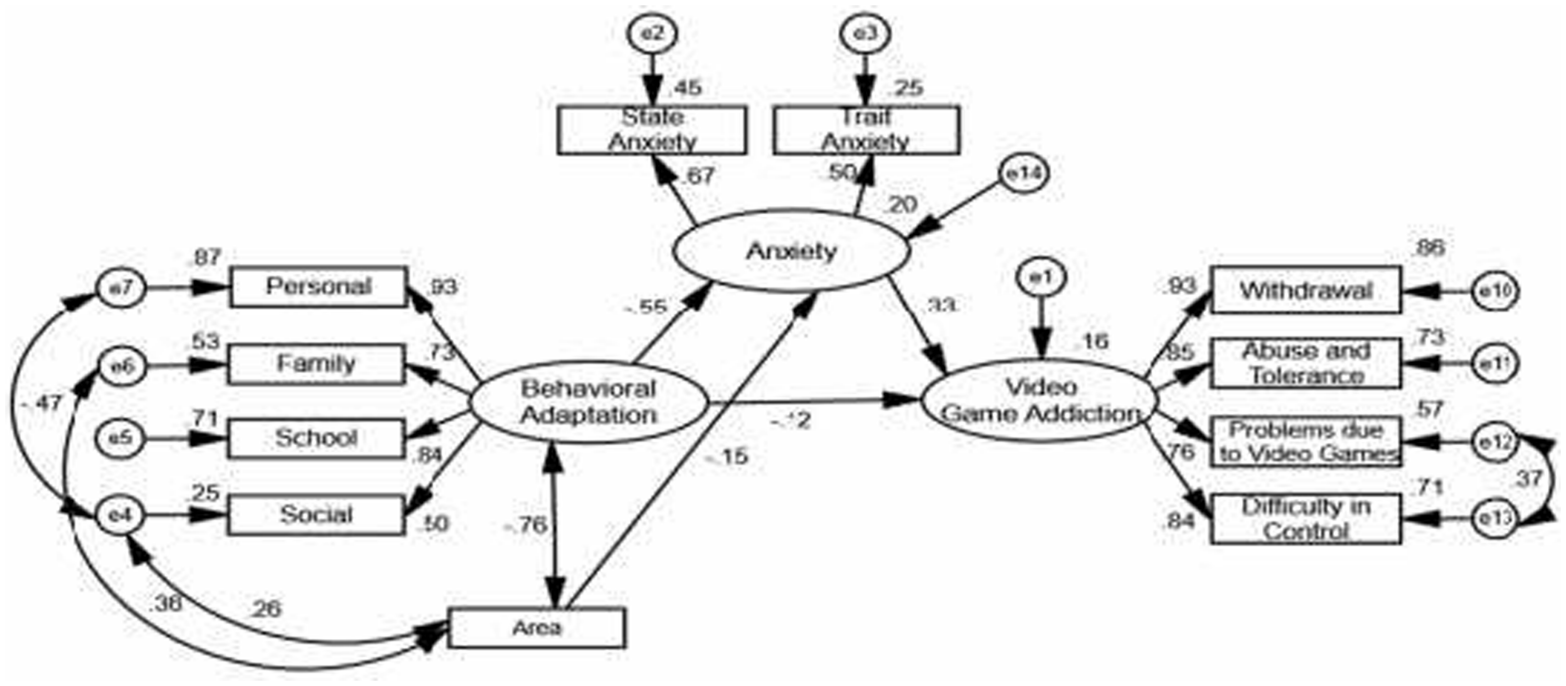
Structural model explaining video game addiction considering the area (urban or rural).

Figure 2, the structural model presented, confirms the proposed hypotheses (H1, H2, H3, and H4), showing that behavioral adaptation and anxiety influence video game addiction, including both direct and indirect effects mediated by anxiety, in adolescents from urban and rural areas. The model fit indices are adequate, with satisfactory values (χ^2^/df = 4.836; TLI = 0.945; CFI = 0.964; GFI = 0.950; RMSEA = 0.080, 90% CI [0.068, 0.092]). It is observed that the standardized coefficients predicting the structural model of video game addiction are as follows: (a) The variable X (Behavioral Adaptation) must have a significant effect on the mediator (Anxiety): X → M (*β* = −0.55; *p* < 0.001), (b) The variable X (Behavioral Adaptation) must have a significant effect on the variable Y (Video Game Addiction): X → Y (*β* = −0.12; *p* < 0.024), and (c) The variable M (Anxiety) must have a significant effect on Y (Video Game Addiction): M → Y (*β* = 0.33; *p* < 0.001).

If the aforementioned conditions (a, b, c) are met, it must also be satisfied that the direct effect (*β* = −0.12) of the independent variable X (Behavioral Adaptation) on the dependent variable Y (Video Game Addiction) is smaller than the indirect effect (*β* = 0.33), which is observed in the model.

**Figure 3:**
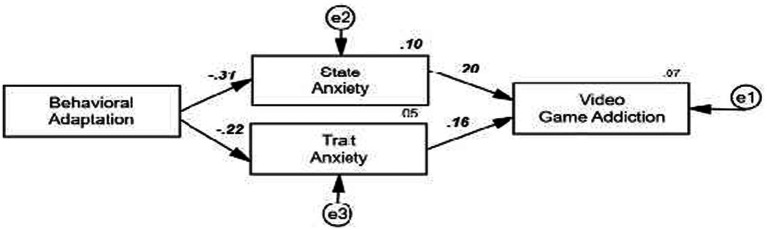
Research hypothesis on the mediating role of anxiety in explaining behavioral adaptation and video game addiction.

Figure 3, shows a mediation model evaluating the effects of state and trait anxiety on the relationship between behavioral adaptation and video game addiction. The results confirm hypothesis H5, demonstrating that state anxiety has a more significant and negative mediating effect than trait anxiety. Specifically, lower behavioral adaptation is associated with an increase in state anxiety (*β* = −0.31), which, in turn, leads to higher levels of video game addiction (*β* = 0.20). Similarly, lower behavioral adaptation is also linked to an increase in trait anxiety (*β* = −0.22), and higher levels of trait anxiety contribute positively to video game addiction (*β* = 0.16). These findings highlight the differential mediating role of both types of anxiety, with state anxiety being the most influential mediating factor in the relationship between behavioral adaptation and video game addiction.

Moreover, behavioral adaptation is associated with the area (*β* = −0.76). Applying the Student’s *t*-test for independent samples [*t*_(604)_ = 17.804; *p* < 0.001; *d* = 1.493] shows that students from rural areas (*M* = 47.4; *SD* = 12.0) exhibit lower behavioral adaptation compared to their urban counterparts (*M* = 68.7; *SD* = 15.5). Similarly, results for state anxiety [*t*_(604)_ = 3.610; *p* < 0.001; *d* = −0.303] and trait anxiety [*t*_(604)_ = 3.341; *p* < 0.001; *d* = −0.280] also indicate that adolescents from rural areas (state anxiety: *M* = 33.8; *SD* = 5.3; trait anxiety: *M* = 34.6; *SD* = 5.4) present higher levels of anxiety than adolescents from urban areas (state anxiety: *M* = 32.2; *SD* = 5.1; trait anxiety: *M* = 32.9; *SD* = 6.1).

## Discussion

The results obtained confirmed the consistency of the proposed structural model, with satisfactory goodness-of-fit indices (χ^2^/df = 4.836; TLI = 0.945; CFI = 0.964; GFI = 0.950; RMSEA = 0.080, 90% CI [0.068, 0.092]). These values support the validity of the model and its capacity to explain the relationships between behavioral adaptation, anxiety, and video game addiction among Peruvian adolescents from urban and rural areas. Based on this foundation, the findings validated the first four hypotheses proposed. Behavioral adaptation showed a significant effect on anxiety (*β* = −0.55, *p* < 0.001), the relationship between behavioral adaptation and video game addiction was confirmed (*β* = −0.12, *p* < 0.024), and the relationship between anxiety and video game addiction was verified (*β* = 0.33, *p* < 0.001), reinforcing its mediating role. Additionally, state anxiety was found to have a greater effect than trait anxiety, highlighting the impact of immediate emotional responses on problematic video game use.

Regarding the sample results, it was observed that the general levels of behavioral adaptation were low, as were the levels of trait anxiety, state anxiety, and video game addiction. Among the dimensions of behavioral adaptation, personal adaptation had the most significant weight (*λ* = 0.93), followed by school adaptation (λ = 0.84). Finally, adolescents from rural areas presented lower levels of behavioral adaptation compared to urban adolescents [*t*_(604)_ = 17.804; *p* < 0.001; *d* = 1.493].

In evaluating the hypotheses, the study confirmed a significant negative relationship between behavioral adaptation and anxiety (*β* = −0.55, H1). This finding suggests that as adolescents’ capacity to adjust to social, emotional, and academic demands improves, anxiety levels decrease. This relationship is consistent with previous research indicating that adaptive difficulties increase anxiety levels, creating emotional vulnerability. Studies such as Li et al. (2023) and Lanfredi et al. (2021) emphasize that adaptive problems amplify psychological distress and hinder stress management. Similarly, Tariq et al. (2021) identified that problematic adaptive schemas exacerbate stress, while Wang et al. (2020) noted that maladaptation contributes to a perpetuating cycle of anxiety and emotional difficulties. These results are particularly relevant during adolescence, a stage characterized by significant adaptive challenges.

Regarding H2, behavioral adaptation showed a direct but low effect on video game addiction (*β* = −0.12), confirming a significant but limited relationship when considered in isolation. This result aligns with the theoretical I-PACE model and various authors such as Dong et al. (2019), Brand (2017), and Király et al. (2023), who emphasize the interaction of multiple factors in the development of addictive behaviors. Although specific research in this context is lacking, prior studies suggest that biopsychological changes during adolescence increase emotional sensitivity and hinder efficient resolution of daily problems (Lin et al., 2024). This may intensify negative thoughts and emotions, motivating adolescents to resort to immediate avoidance strategies such as excessive video game use (Von der Heiden et al., 2019; Estévez et al., 2021; Lin et al., 2024).

Anxiety demonstrated a significant correlation with video game addiction (*β* = 0.33), reinforcing its role as a critical factor in the formation of addictive behaviors and confirming H3. This finding is consistent with previous studies identifying anxiety as a key predictor of problematic video game use as an emotional regulation strategy (Li et al., 2023). Wang et al. (2020) found a positive relationship between social anxiety and video game addiction, while Qin et al. (2023) also reported a positive correlation between these variables, although this was not the primary focus of their analysis.

Regarding H4, the analysis confirmed that anxiety acts as an indirect mediating effect in the relationship between behavioral adaptation and video game addiction, amplifying the impact of adaptive difficulties on the development of addictive behaviors. This finding supports the idea that video game addiction depends on a complex interaction of multiple factors, as highlighted in studies by Dong et al. (2019) and Király et al. (2023).

Anxiety in general this relationship by manifesting at different levels during addiction formation. As a personal factor, trait anxiety reflects a stable predisposition toward negative emotions, increasing the likelihood that adolescents use video games as a coping strategy (Von der Heiden et al., 2019; Fujihara et al., 2023). From an affective and cognitive perspective, anxiety intensifies threat perception and hampers emotional regulation, promoting avoidance patterns that contribute to problematic video game use (Wang et al., 2020; Qin et al., 2023). Additionally, elevated anxiety states can interfere with executive functions, affecting key skills such as attention, cognitive flexibility, and decision-making (Neo et al., 2011; Moran, 2016; Hubbard et al., 2020; Dolz-del-Castellar and Oliver, 2021). These limitations hinder the evaluation of long-term consequences, reinforcing compulsive behaviors that perpetuate dependence (Von der Heiden et al., 2019; Huang et al., 2022).

In terms of the difference between anxiety types, the results align with the final hypothesis (H5), demonstrating that state anxiety has a stronger mediating effect than trait anxiety.

It could be that the reason why state anxiety has a more significant effect as a mediator in video game addiction is related to how each type of anxiety behaves and the effects it produces at the brain level (Wiers and Wiers, 2016; Billieux et al., 2017; Yang et al., 2018). Thus, the first key difference found lies in the motivation that triggers anxiety (Eysenck et al., 2007; Zhou and Siu, 2015; Wiers and Wiers, 2016). While trait anxiety is related to a permanent perception of threats in the environment, state anxiety is associated with a specific threat that could even be the reason for its emergence (Dolz-del-Castellar and Oliver, 2021; Fujihara et al., 2023; Portela-Pino and Dominguez Rodriguez, 2023).

To better understand it, it is necessary to understand fear, a variable closely associated with anxiety that imparts a negative emotional intensity (Gawda and Szepietowska, 2016; Yang et al., 2018; Toh and Yang, et al., 2020). Thus, trait anxiety is linked to a diffuse fear (Öhman, 2008); in this type of anxiety, individuals experience a sense of insecurity caused by an unspecified fear or several minor fears (Fujihara et al., 2023; Portela-Pino and Dominguez Rodriguez, 2023). Conversely, state anxiety is directed toward a specific situation or problem (Dolz-del-Castellar and Oliver, 2021; Fujihara et al., 2023; Portela-Pino and Dominguez Rodriguez, 2023). Although negative emotions are present in both forms of anxiety, they differ: in trait anxiety, these emotions are less intense but more enduring, while in state anxiety, they are temporary but intense (Pacheco-Unguetti et al., 2010; Torrano et al., 2020). This intensity could represent one of the keys to the development of addiction.

Additionally, another important difference for the study could be the inhibition process (Neo et al., 2011; Martin-Ramos et al., 2015; Yang et al., 2018; Toh and Yang, 2020). Although both types of anxiety affect inhibition, the way they do so might be related to the results of this study. Trait anxiety, being persistent, appears to generate brain-level modifications that induce a constant focus on threats, inhibiting other attentional processes (Finucane, 2011; Martin-Ramos et al., 2015; Toh and Yang, 2020). In this context, trait anxiety is associated with sustained activation of brain structures involved in emotional regulation, such as the amygdala and prefrontal cortex, which promotes continuous alertness to perceived dangerous stimuli, with difficulty in modulating this response (Bishop, 2009; Neo et al., 2011; Yang et al., 2018; Toh and Yang, 2020).

On the other hand, state anxiety, in its initial phase, affects proactive cognitive control, which can lead to indecision or difficulty initiating an action, similar to trait anxiety at this stage (Yang et al., 2018). However, individuals with state anxiety, although they may have difficulties in inhibiting attention before confronting the situation or problem, can focus their attention better when directly facing anxiety, neutralizing distractions and concentrating on the perceived threat (Finucane, 2011; Pacheco-Unguetti et al., 2010; Yang et al., 2018; Toh and Yang, 2020). This process is related to reactive cognitive control, which proves to be more effective in this context (Yang et al., 2018).

Therefore, it could be postulated that during a state anxiety episode, the individual has the biological capacity to concentrate on a single focus of attention, which, in an appropriate adaptation process, would be the specific problem (Eysenck et al., 2007; Yang et al., 2018). In contrast, individuals with trait anxiety have difficulty focusing on a single stimulus, as their hypersensitivity to emotional and cognitive stimuli prevents them from concentrating their attention, favoring the spread of attention to multiple sources of distraction, both positive and negative (Pacheco-Unguetti et al., 2010; Yang et al., 2018; Toh et al., 2020).

In relation to this study, it is relevant to consider how each type of anxiety acts as a means of escape, taking into account both emotion and inhibition (Carbonell et al., 2017; Billieux et al., 2017; Chen et al., 2023). State anxiety could be more influential, as adolescents with adaptation problems, amplified by anxiety, would not use their problem-solving skills to focus on the situation but instead shift their attention to symbolic situations, such as video games, which offer a problem-solving experience (Von der Heiden et al., 2019). Some studies support this idea through their neural analyses related to video game use (Gray and McNaughton, 2000; Roy et al., 2021; Chen et al., 2023).

Considering the importance of dopamine-generating emotionality and how it has a key impact on addictive memory (Zhou and Siu, 2015; Wiers and Wiers, 2016; Chen et al., 2023; Xu et al., 2024), it is essential to understand the brain processes involved. State anxiety would have a greater impact on video games due to its pronounced intensity (Zhou and Siu, 2015), as it would more strongly activate brain areas involved in reward and motivation, such as the dopaminergic system (Wiers and Wiers, 2016; Palaus et al., 2017; Reynolds and Flores, 2021). Unlike trait anxiety, which is less intense but more frequent, generating consistency in gaming behavior and potentially facilitating the formation of addiction. However, as proposed by Brand (2017), no single variable predicts addiction; rather, it is a combination of factors. This study, with its limitations, does not aim to offer a definitive conclusion but seeks to move closer to a broader understanding of this phenomenon.

Additionally, these assumptions are supported by prior research, such as Mehroof and Griffiths (2010), who suggest that this could be due to the intensity of emotions involved. Other studies also provide relevant evidence (Huerta et al., 2020; De Pasquale et al., 2021; Dolz-del-Castellar and Oliver, 2021; Fujihara et al., 2023; Mishu et al., 2023). However, more specific studies are suggested, as most current research focuses on anxiety in general without specifying differences between its types.

The low levels of behavioral adaptation observed in the sample may reflect a context in which adolescents lack effective skills to manage their environment, contributing to greater dependence on video games. The strength of the coefficients found in adaptation dimensions (personal adaptation: *λ* = 0.93; school adaptation: *λ* = 0.84) highlights the importance of individual skills and the school environment as key determinants of behavioral and emotional adjustment (Wang et al., 2020; Méndez et al., 2021; Lin et al., 2024).

In this regard, Méndez et al. (2021) indicate that, within the adaptation process, family adaptation is one of the most important as it provides the foundation for other adaptations. However, in the context of adolescents, school adaptation may acquire special relevance (Choe et al., 2024; Alvarez and Castelló, 2020), considering that recognition from teachers and peers constitutes an important means of validation (Alvarez and Castelló, 2020; Gutiérrez et al., 2021). On the other hand, authors such as Santander et al. (2022) and Maya et al. (2023) point out that adaptation is closely related to social support in a bidirectional relationship, suggesting that low levels of adaptation could negatively impact adolescents’ perception of support (Gutiérrez et al., 2021), generating adverse effects on their well-being (Lin et al., 2024; Ruiz and Yabut, 2024). Méndez et al. (2021) also highlight that personal adaptation results from prior adaptations such as family, social, and academic adaptations. Thus, in the analyzed sample, results indicating low adaptation suggest the need for interventions in school and personal domains.

Finally, the differences observed between adolescents from rural and urban areas underscore the influence of context on behavioral adaptation (Choe et al., 2024). Rural adolescents, presenting lower levels of adaptation, may face greater challenges in accessing educational and social resources, increasing their vulnerability to problematic behaviors such as video game addiction. In this regard, Sánchez-Castro et al. (2024) and Choe et al. (2024) indicate that socioeconomic and opportunity differences significantly influence the proper development of children and adolescents, as the stress factors associated with contextual deficiencies hinder the optimal development of their skills (Choe et al., 2024). This may be related to the results of this study, considering that the rural population in Peru is vulnerable both economically and geographically (UNICEF, 2021; Cavagnoud, 2023).

From a theoretical perspective, the results of this study provide significant evidence about the underlying dynamics in the relationship between behavioral adaptation, anxiety, and video game addiction in adolescents. The findings highlight the central role of anxiety as a key mediator, reinforcing theories emphasizing the interaction between emotional and behavioral factors in the formation of problematic behaviors. Additionally, the identification of state anxiety as a more influential mediating factor than trait anxiety offers a new perspective for understanding how emotional and situational responses, rather than stable emotional predispositions, can intensify addictive behaviors in specific contexts. These contributions enrich the existing theoretical body by demonstrating the importance of addressing both adaptive difficulties and fluctuating emotional states in the development of behavioral addictions during adolescence, especially in vulnerable socio-educational contexts.

From a practical perspective, the results of this study underscore the importance of interventions aimed at preventing video game addiction in adolescents, especially those from rural areas where lower levels of behavioral adaptation were found. This finding reinforces the need for strategies that strengthen individual competencies and school dynamics in contexts with limited access to educational and social resources, as low behavioral adaptation is a predisposing factor for problematic behaviors. Although trait and state anxiety levels were moderately low in the sample, their mediating role in the relationship between behavioral adaptation and video game addiction highlights the importance of addressing emotional distress that can act as a trigger for problematic video game use. Additionally, the low levels of video game addiction observed in the sample offer a favorable framework for implementing preventive programs that consolidate socio-emotional skills, preventing adolescents from resorting to this form of entertainment as an avoidance strategy. These practical implications suggest that interventions should focus not only on improving behavioral adaptation but also on addressing the emotional and contextual factors that increase vulnerability to video game addiction.

Although this study provides a comprehensive perspective on the relationship between behavioral adaptation, anxiety, and video game addiction among Peruvian adolescents, it presents some limitations that should be considered. First, the cross-sectional design limits the ability to establish definitive causal relationships between the variables studied. While the SEM model allows exploring direct and indirect relationships, the data obtained represent a specific moment in time, preventing observation of how these interactions might evolve longitudinally.

Second, the use of non-probabilistic convenience sampling could limit the generalization of the findings to other adolescent populations, particularly in different cultural or socioeconomic contexts. Although the sample included adolescents from urban and rural areas, differences in access to educational and social resources between these contexts could influence the interpretation of the results.

Additionally, although the structural model’s fit indices were satisfactory, the moderate correlations observed between the variables suggest the need to include other relevant factors, such as family dynamics or the school environment, to obtain a more complete view of the factors contributing to video game addiction. Finally, although the instruments used showed acceptable validity and reliability in this sample, future research could consider broader validation of these instruments in similar contexts to ensure robustness.

These limitations underscore the importance of conducting future research with longitudinal designs and more representative samples, as well as including additional variables that allow for a deeper analysis of the factors affecting video game addiction in adolescents.

## Data Availability

The raw data supporting the conclusions of this article will be made available by the authors, without undue reservation.
